# Supplementary material for: Gender differences in provider practice characteristics and medicare payment & services among diagnostic radiologists
Source: PLoS One. 2026 Jun 5;21(6):e0349413. doi: 10.1371/journal.pone.0349413 (PMC13240891; doi:10.1371/journal.pone.0349413)
Supplement: Attachment — Submitted filename: PLOS Reviewers.docx [file pone.0349413.s002.docx]

Reviewers' comments:

Reviewer's Responses to Questions

**Comments to the Author**

1. Is the manuscript technically sound, and do the data support the conclusions?

The manuscript must describe a technically sound piece of scientific research with data that supports the conclusions. Experiments must have been conducted rigorously, with appropriate controls, replication, and sample sizes. The conclusions must be drawn appropriately based on the data presented.

Reviewer #1: Yes

Reviewer #2: Partly

**Response: We have added to the analysis as suggested.**

2. Has the statistical analysis been performed appropriately and rigorously?

Reviewer #1: Yes

Reviewer #2: No

**Response: We have added to the statistical analysis as suggested and performed multivariable regression- this has been added to the Methods, Results and Discussion sections.**

3. Have the authors made all data underlying the findings in their manuscript fully available?

The [PLOS Data policy](https://nam12.safelinks.protection.outlook.com/?url=http%3A%2F%2Ftrack.editorialmanager.com%2FCL0%2Fhttp%3A%252F%252Fwww.plosone.org%252Fstatic%252Fpolicies.action%2523sharing%2F1%2F010f01974494fd36-41c47a65-2f48-4b29-b7c8-1ce8186df265-000000%2FDC87Sp6d-0jcMlxpf7oiL-3TH6srp_yBvACWripYkuo%3D214&data=05%7C02%7Cajay.malhotra%40yale.edu%7C62dd9dd3a6e3419e2f9708dda4dcf401%7Cdd8cbebb21394df8b4114e3e87abeb5c%7C0%7C0%7C638847991398362547%7CUnknown%7CTWFpbGZsb3d8eyJFbXB0eU1hcGkiOnRydWUsIlYiOiIwLjAuMDAwMCIsIlAiOiJXaW4zMiIsIkFOIjoiTWFpbCIsIldUIjoyfQ%3D%3D%7C0%7C%7C%7C&sdata=oy93vkRookqta5F7LFkD9LXkspHBFA3tmmaJR6q0zO4%3D&reserved=0) requires authors to make all data underlying the findings described in their manuscript fully available without restriction, with rare exception (please refer to the Data Availability Statement in the manuscript PDF file). The data should be provided as part of the manuscript or its supporting information, or deposited to a public repository. For example, in addition to summary statistics, the data points behind means, medians and variance measures should be available. If there are restrictions on publicly sharing data—e.g. participant privacy or use of data from a third party—those must be specified.

Reviewer #1: No

Reviewer #2: Yes

**Response: We have now added the Data Availability Statement and clarified that the data is publicly available and provided the source.**

4. Is the manuscript presented in an intelligible fashion and written in standard English?

PLOS ONE does not copyedit accepted manuscripts, so the language in submitted articles must be clear, correct, and unambiguous. Any typographical or grammatical errors should be corrected at revision, so please note any specific errors here.

Reviewer #1: Yes

Reviewer #2: Yes

5. Review Comments to the Author

Please use the space provided to explain your answers to the questions above. You may also include additional comments for the author, including concerns about dual publication, research ethics, or publication ethics. (Please upload your review as an attachment if it exceeds 20,000 characters)

Reviewer #1: The manuscript presents an original and timely analysis of gender-based disparities in practice characteristics and Medicare payments among diagnostic radiologists. The topic is highly relevant, and the analysis is based on a large, publicly available dataset, with appropriate statistical comparisons. The manuscript is technically sound, and the conclusions are well-supported by the data. However, I recommend minor revisions to address key clarifications and align with PLOS ONE’s standards for reproducibility and transparency.
Abstract: On Line 28, correct “differences among radiologists is poorly understood” to “are poorly understood.” On Lines 57–60, clarify the payment comparison: “female radiologists received 86% of the total Medicare payments received by male radiologists.” Remove space before semicolons in statistical reporting (e.g., Line 55: “9,859 ;” → “9,859;”). This line is a little confusing, please fix this.

**Response: We have now incorporated the suggestions and made corrections.**

Methods (Lines 91–141): The methodology is appropriate, but a few key variable definitions are missing. Please clarify:
How gender was assigned (e.g., from NPPES or another linked source) — not specified in current version.
How academic vs. non-academic status was defined (Line 112 onward).
How urban vs. rural practice was classified (Line 114).
How practice size was measured (Line 116).

**Response: We have now incorporated the suggestions and clarified the definition sources.**

Line 120 contains a typo: correct “breath of practice” to “breadth of practice.” On Line 133, clarify whether a two-tailed alpha level (e.g., α = 0.05) was used and if any R packages were applied in analysis.
Results (Lines 142–191): The results are clearly presented and statistically robust. To improve clarity:
On Lines 158–161, rephrase for interpretability: “34.2% of academic radiologists were female, compared to 22.6% in non-academic settings.”
On Line 164, clarify that 64.9% refers to female representation within breast imaging.

**Response: We have now incorporated the suggestions and made corrections.**

On Lines 142–145, specify how p-values for time trends were calculated (e.g., test for trend vs simple comparison).

**Response: We have now incorporated the suggestion and added to Methods and Results. Trends in payment amounts over time were determined with a time-series analysis with a two-tailed alpha level (α = 0.05).**

For statements like “per-service payments were similar within subspecialties” (Line 172), consider adding whether statistical testing confirmed this or clarify as an observation.

**Response:** **We have now incorporated the suggestion.**

Discussion (Lines 192–247): Well-structured and grounded in existing literature. On Line 193, fix heading typo: “DICUSSION” → “DISCUSSION.” Line 184 contains a grammatical error: “the a prior study” → “a prior study.” On Line 159, temper speculation: replace “will plateau” with “may plateau.” Similarly, Line 107–110 speculates about non-Medicare income sources — please clearly frame this as a hypothesis. Consider briefly discussing implications or areas for future research (e.g., understanding drivers of volume differences).

**Response: We have now incorporated the suggestions and made corrections.**

Limitations (Lines 248–266): Strong and transparent. On Line 252, rephrase “only <0.1%” to “fewer than 0.1%.” Consider noting that working hours or FTE status were not available and could influence payment differences. Also mention that multivariable adjustment was not performed.

**Response: We have now incorporated the suggestions and made corrections.**

Conclusion (Lines 263–266): Appropriate and data-aligned. Line 264 may read better as: “payments per service were higher for female radiologists than for males overall, but were comparable between genders within the same subspecialty.”

**Response: We have now incorporated the suggestions and made corrections.**

Data Availability (Lines 93–95, 267–268): Current statement (“CMS Public files”) is insufficient. Please expand to include dataset name, years used, and a direct URL (e.g., “https://data.cms.gov/...”).

**Response: We have now incorporated the suggestions and made corrections.**

Minor Style Issues: Maintain consistent terminology (e.g., “female/male radiologists”), fix small typos, and add table/figure cross-references where appropriate in Results.
In summary, this is a strong manuscript that requires minor revisions to address transparency, clarify definitions, and polish a few points of language and formatting. Once addressed, it will meet the publication standards of PLOS ONE.

Reviewer #2: In the study by Malhotra et al., the authors sought to compare Medicare payments between female and male diagnostic radiologists. Overall, this is an interesting study and is well written. I have a major concern:

Why did the authors not conduct a multivariate regression to see an independent association between and medicare payments? From the results, it is seen that female radiologists are more likely to be in academic practice compared to male radiologists. The practice type could be a confounder/ effect modifier and therefore, we cannot draw conclusions without multivariate analysis.

**Response: We have now incorporated the suggestions and performed multivariate regression- this has been added to the Methods, Results and Discussion sections.**

6. PLOS authors have the option to publish the peer review history of their article ([what does this mean?](https://nam12.safelinks.protection.outlook.com/?url=https%3A%2F%2Ftrack.editorialmanager.com%2FCL0%2Fhttps%3A%252F%252Fjournals.plos.org%252Fplosone%252Fs%252Feditorial-and-peer-review-process%2523loc-peer-review-history%2F1%2F010f01974494fd36-41c47a65-2f48-4b29-b7c8-1ce8186df265-000000%2F6gMS8MaFoMRRcBk6zVxgv-wQ0PMkqfMHRyD14JRhGkE%3D214&data=05%7C02%7Cajay.malhotra%40yale.edu%7C62dd9dd3a6e3419e2f9708dda4dcf401%7Cdd8cbebb21394df8b4114e3e87abeb5c%7C0%7C0%7C638847991398375896%7CUnknown%7CTWFpbGZsb3d8eyJFbXB0eU1hcGkiOnRydWUsIlYiOiIwLjAuMDAwMCIsIlAiOiJXaW4zMiIsIkFOIjoiTWFpbCIsIldUIjoyfQ%3D%3D%7C0%7C%7C%7C&sdata=4A4O%2BuqUgee4%2BT9QGtsvEUzDbVbUyDr3Z7U1%2BUgSbh0%3D&reserved=0)). If published, this will include your full peer review and any attached files.


If you choose “no”, your identity will remain anonymous but your review may still be made public.


**Do you want your identity to be public for this peer review?** For information about this choice, including consent withdrawal, please see our [Privacy Policy](https://nam12.safelinks.protection.outlook.com/?url=https%3A%2F%2Ftrack.editorialmanager.com%2FCL0%2Fhttps%3A%252F%252Fwww.plos.org%252Fprivacy-policy%2F1%2F010f01974494fd36-41c47a65-2f48-4b29-b7c8-1ce8186df265-000000%2FDLD4DDFUZYjfnBXaFlYpYYKVX0z-HrP4-Vp4mgsanBE%3D214&data=05%7C02%7Cajay.malhotra%40yale.edu%7C62dd9dd3a6e3419e2f9708dda4dcf401%7Cdd8cbebb21394df8b4114e3e87abeb5c%7C0%7C0%7C638847991398388718%7CUnknown%7CTWFpbGZsb3d8eyJFbXB0eU1hcGkiOnRydWUsIlYiOiIwLjAuMDAwMCIsIlAiOiJXaW4zMiIsIkFOIjoiTWFpbCIsIldUIjoyfQ%3D%3D%7C0%7C%7C%7C&sdata=giZEBOPln7gRHhZIs8THiRHiCD9ZULin1b7oL7CIUfY%3D&reserved=0).

Reviewer #1: **Yes:** N/A

Reviewer #2: No
