# Supplementary material for: Gender differences in provider practice characteristics and medicare payment & services among diagnostic radiologists
Source: PLoS One. 2026 Jun 5;21(6):e0349413. doi: 10.1371/journal.pone.0349413 (PMC13240891; doi:10.1371/journal.pone.0349413)
Supplement: Attachment — Submitted filename: ResponsetoReviewCL_MKedits.docx [file pone.0349413.s003.docx]

6. Review Comments to the Author

Please use the space provided to explain your answers to the questions above. You may also include additional comments for the author, including concerns about dual publication, research ethics, or publication ethics. (Please upload your review as an attachment if it exceeds 20,000 characters)

Reviewer #3: Doctors --

OVERALL

This manuscript addresses an interesting, policy-relevant question; the CMS Physician/Other Practitioners public files are a reasonable source for a descriptive look at radiology workload and Medicare payments.

Your headline findings: women are ~25% of diagnostic radiologists; more likely to be academic, urban, in large groups, and in certain subspecialties (notably breast); women have lower total Medicare payments but higher payment per service overall, with parity within the same subspecialty. All of that is clearly stated.

RECOMMENDATIONS

1. Adjust beyond bivariate tests.

Right now, means/medians are compared with t-tests/Wilcoxon. That’s not enough for a question this confounded (years in practice, region, urbanicity, practice size, subspecialty mix, year). Add multivariable models (e.g., panel OLS/GLM with radiologist and year fixed effects or at least covariate adjustment with clustered SEs by NPI).

Report adjusted differences and CIs.

Response: Thank you for your comment. In our analysis, we aggregated the data at the NPI level for each individual calendar year, such that each observation represents a physician-year. While this structure allows for repeated observations of the same physician across years, practice patterns may vary meaningfully over time (e.g., due to sabbaticals, transitions to part-time work, or retirement). Because these factors cannot be reliably captured in the dataset, we did not consider it appropriate to pool observations across years at the physician level.

Also, we do not observe substantial year-to-year variations, and the calendar year was not considered a significant confounder. Accordingly, we utilized unadjusted comparisons (t-tests and Wilcoxon rank-sum tests) to evaluate differences between groups.

2. Decompose “payment per service.”

Your own discussion concedes this metric reflects code mix, professional vs global billing, facility vs non-facility, and regional GPCI, not “pay” per se. Do a mix-adjustment: (i) show the top CPT/HCPCS distributions by sex; (ii) reweight to a common code mix; (iii) report a case-mix–adjusted payment per service. That will test whether the 18% higher female PPS is mix, geography, or true per-code differences (which should be zero under the fee schedule).

Also break out professional-only vs global where feasible.

Response: We agree that the observed difference in payment per service likely reflects variations in code mix, billing composition, and geographic factors. While a formal case-mix adjustment falls outside the macro-level scope of our study, Table 1 outlines broad distributional differences in practice composition by gender. To address this important point, we have added a sentence to the Discussion clarifying that this metric is driven by differences in subspecialty and modality mix.

3. Subspecialty classification transparency.

You infer subspecialty by >50% wRVUs using NITOS modality/body-region mapping. Clever idea — recommend to document it fully in the Methods (code lists, thresholds, sensitivity with 60%/70% cutoffs). Right now the summary references prior validation but your implementation details (exact crosswalks) need to be reproducible. Deposit the mapping.

Response: Thank you for your comment. We will document it in the Methods. This threshold and classification strategy were derived from the methodology described by Rosenkrantz et al. (J Am Coll Radiol, 2017), which validated a claims-based approach to subspecialty assignment using Medicare data. We adapted their approach by applying the same >50% wRVU threshold to assign each radiologist a single dominant subspecialty based on their distribution of billed services.

Rosenkrantz AB, Wang W, Hughes DR, Ginocchio LA, Rosman DA, Duszak R Jr. Academic Radiologist Subspecialty Identification Using a Novel Claims-Based Classification System. AJR Am J Roentgenol. 2017 Jun;208(6):1249-1255. doi: 10.2214/AJR.16.17323. Epub 2017 Mar 16. Erratum in: AJR Am J Roentgenol. 2017 Aug;209(2):472. doi: 10.2214/AJR.17.18609. PMID: 28301213.

Rosenkrantz AB, Wang W, Bodapati S, Hughes DR, Duszak R Jr. Private Practice Radiologist Subspecialty Classification Using Medicare Claims. J Am Coll Radiol. 2017 Nov;14(11):1419-1425. doi: 10.1016/j.jacr.2017.04.025. Epub 2017 Jun 30. PMID: 28673776.

4. Define “academic vs non-academic,” “urban vs rural,” and “years in practice.”

You cite practice location and NPPES for “years in practice,” but the operational definitions aren’t spelled out (RUCA vs MSA? Years since NPI enumeration != years in practice). Add precise algorithms and references.

Response: Thank you for your comment. We have clarified the operational definitions in the Methods section. The academic status of practices associated with radiologists in the CMS files was obtained from the Harvey L. Neiman Health Policy Institute. Per the methodology provided by the institute, practices were classified as academic if their names contained terms such as “university,” “faculty,” “college,” or “school,” and were further validated through manual review against the list of diagnostic radiology residency programs provided by the AAMC. Urban versus rural status was determined using Rural-Urban Commuting Area (RUCA) codes based on practice ZIP code. “Years in practice” was derived from the physician’s medical school graduation year as reported in the publicly available National Plan & Provider Enumeration System (NPPES) database and linked via NPI.

5. Repeated measures / clustering.

Are observations pooled across 2017–2021 at the physician-year level? If so, your tests must account for within-physician correlation across years (clustered SEs) and secular trends (year fixed effects). The time-trend figure suggests a panel; analyze it as such.

Response: Thank you for this comment. Our dataset is structured at the physician-year level, with each observation representing a unique NPI in a given calendar year. While this allows for repeated observations of the same physician across years, we did not model the data as a longitudinal panel. This decision was based on the potential for meaningful year-to-year variation in individual practice patterns (e.g., sabbaticals, transitions to part-time work, or retirement), which cannot be reliably captured in the dataset.

Also, we do not observe substantial year-to-year variations, and the calendar year was not considered a significant confounder. Accordingly, we utilized unadjusted comparisons (t-tests and Wilcoxon rank-sum tests) to evaluate differences between groups.

6. Tables contain obvious inconsistencies.

In Table 1, the “All years” column shows tiny denominators (e.g., “Academic 34.2% (221/948)”) that don’t match the reported overall N (33,029). Audit and correct these lines before anything else. Similar spot-checks across Table 3 are prudent.

Response: The denominators in Table 1 reflect only physicians with available data for the specified variable (e.g., practice type. As such, we excluded analysis like “Academic vs Non Academic” for physicians who did not have data available.

7. Terminology and source for “gender.”

State explicitly how “gender” is obtained in CMS files (binary sex field, self-report, inference?). Use consistent terminology and acknowledge limitations (non-binary not captured).

Response: Thank you for your comment. Gender was obtained from the CMS dataset as a self-reported, binary variable (male/female). We have clarified this in the Methods section and now acknowledge this as a limitation, as non-binary gender identities are not captured in the dataset.

8. Scope the inference carefully.

You study FFS Medicare claims only. Don’t imply salary conclusions or “pay gaps”; these are Medicare reimbursements, not total compensation, and exclude Medicare Advantage/commercial. You mention this in Limitations—bring that caution forward into the Abstract and Conclusions.

Response: Thank you for your comment. We agree that our inferences should be carefully scoped to reflect that the analysis is based on FFS Medicare claims data and does not represent total physician compensation. We have detailed this in the limitations section and added a sentence about this in the discussion.

9. Data/code availability.

In the spirit of replicability: provide exact dataset names/years/URLs and deposit all code and crosswalks (wRVU/NITOS subspecialty assignment, urban/rural rules, academic flag) in a public repo. The current data statement should point precisely to those sources.

Response: Thank you for your comment. All data used in this study are publicly available, and specific references to the datasets, including the CMS Medicare Fee-for-Service Provider Utilization and Payment Data (2017–2021) and the National Plan & Provider Enumeration System (NPPES), are provided in the Methods section and references. The code is available from the authors upon request.

MINOR NOTES

- Use rates per radiologist-year (or per 1,000 services) in the text instead of raw totals; keep totals to the tables.

- Report both means and medians for skewed financial variables.

- Tighten typos (e.g., “DICUSSION”), and standardize style in tables/figures.

Thank you for your comment.
